# Clinical results of Intraductal Meibomian gland probing combined with intense pulsed light in treating patients with refractory obstructive Meibomian gland dysfunction: a randomized controlled trial

**DOI:** 10.1186/s12886-019-1219-6

**Published:** 2019-10-28

**Authors:** Xiaodan Huang, Qiyu Qin, Linping Wang, Jiao Zheng, Lin Lin, Xiuming Jin

**Affiliations:** Eye Center, Affiliated Second Hospital, School of Medicine, Zhejiang University, 88 Jiefang Road, Hangzhou, 310009 China

**Keywords:** Intraductal meibomian gland probing, Intense pulsed light, Meibum, Tear film, Obstructive meibomian gland dysfunction

## Abstract

**Background:**

This study aims to optimize the therapeutic regimen for refractory obstructive meibomian gland dysfunction (o-MGD) patients by combining intraductal meibomian gland probing (MGP) and intense pulsed light (IPL) to enhance their positive effects and reduce their limitations.

**Methods:**

This randomized, assessor blind study includes 45 patients (90 eyes) with refractory o-MGD who were divided into 3 groups via allocation concealment: IPL (group I, received an IPL treatment course: 3 times at 3-week intervals), MGP (group II, received MGP one time), and combined MGP-IPL (group III, MGP first followed by an IPL treatment course). Standard Patient Evaluation of Eye Dryness score (SPEED), tear break-up time (TBUT), corneal fluorescein staining (CFS), meibum grade, and lid margin finding results were assessed at baseline, 3 weeks after final treatment for groups I and III, 3 and 12 weeks after MGP for group II. Six months after final treatment, the SPEED and willingness to receive any treatment again were also collected for all groups. Paired Wilcoxon, Mann-Whitney U with Bonferroni correction, and Kruskal-Wallis tests were used for data analysis.

**Results:**

For all 3 groups, all previously mentioned indexes improved significantly following treatment (P<0.01). MGP-IPL was better than IPL and MGP in terms of post-treatment SPEED, TBUT, meibum grade, and lid telangiectasia (P<0.05/3). Furthermore, the MGP-IPL was better than IPL in terms of lid tenderness and better than MGP in terms of orifice abnormality (P< 0.05/3). Six months later, the SPEED for the MGP-IPL was also significantly lower than other groups (P<0.05/3). Moreover, no patients in the MGP-IPL group expressed the need to be treated again compared to 35.7% or 20% of patients in the IPL or MGP groups, respectively.

**Conclusions:**

Compared with IPL or MGP alone, the combination MGP-IPL produced best results in relieving all signs and symptoms and helping patients attain long-lasting symptom relief.

**Trial registration:**

http://clinicaltrials.gov, ChiCTR1900021273 (retrospectively registered February 9, 2019).

## Background

Dry eye has always being considered as a significant health concern that threatens individuals’ life quality as well as their personal and economic well-being [1, 2]. Among various types of dry eye diseases, obstructive meibomian gland dysfunction (o-MGD) causing evaporative dry eye has attracted the attention of clinicians and scientists for its chronic course, recurrent potential, and high incidence rate [3, 4]. Moreover, the obstruction of the terminal tract of the meibomian gland (MG) leads to hyposecretion and quality change of meibum from the orifices [5]. These changes of meibum in ocular surfaces can result in instability of the tear film as well as irritation symptoms such as dryness and foreign body sensation [3]. Additionally, unusually elevated intraglandular pressure and aggravated local inflammation caused by meibum stasis further exacerbate the disease course, creating a vicious cycle.

Traditional treatments for o-MGD include warm compress, massage, artificial tears, etc. However, studies have showed that these treatments are not sufficient for symptom relief [6, 7]. And it is difficult for patients to comply with continuous medical therapies. Chinese o-MGD patients, in particular, always meet serious initial symptoms with MG orifices obstruction and no meibum secretion, making the treatment processes even more difficult [8, 9]. In recent years, great strides have been made in terms of new treatment options for refractory o-MGD patients, one of which is intense pulsed light (IPL). IPL, which has long been used in medical cosmetology, can also be effective for dry eye treatment mainly due to its inhibition of telangiectasias along the eyelid that block the way of inflammatory cytokine and its heating effects [10, 11]. Another relatively new method is intraductal meibomian gland probing (MGP), which was first described by Maskin in 2010. MGP uses a special meibomian cannula to probe the plugged meibomian gland, releasing abnormal elevated intraductal pressure and reestablishing a healthy microenvironment favoring the growth of MG tissues [12].

Although the safety and effectiveness of IPL and MGP have been proven in previous studies [8, 10, 11, 13, 14], their deficiencies can also be observed through day-to-day clinical observation. Specifically, the effect of IPL in alleviating stubborn intraductal congestion or intraductal scarring is comparatively limited. And for patients with severe intraductal inflammation or apparent blepharitis, the use of MGP alone is insufficient for decreasing excessive inflammation. Besides, probing is an invasive method for patients. Sik Sarman et al. reported that 20% of patients require repeated probing after an average of 4.6 months [13]. Repeated Probing may bring psychological burden to patients and would possibly cause scar proliferation. It is thus an urgent matter to identify an optimal therapeutic regimen that can reduce the number of invasive treatments, open the MG obstruction, promote the discharge of meibum, and at the same time, control inflammation.

Here, a new treatment method that combined the MGP and IPL courses was devised and then compared with MGP, IPL alone, with the aim of identifying a way in which to strengthen the advantages of MGP and IPL, and at the same time, offset their side-effects. All participating patients had serious refractory o-MGD and more than half of their evaluated meibomian gland orifices obstructed with no lipid secretion. Additionally, their Meibo-Scans showed no extensively atrophied areas.

## Methods

This randomized controlled, assessor blind study was conducted between July 1, 2018 and December 30, 2018.

### Patient selection and study design

45 patients clinically diagnosed with refractory o-MGD enrolled in this study. The inclusion criteria included: (1) older than 18 years, (2) Standard Patient Evaluation of Eye Dryness (SPEED) questionnaire≥6, (3) more than half of the 15 evaluated meibomian gland orifices in each eyelid were obstructed and had no lipid secretion with extrusion, (4) meibum grade ≤ 24, (5) breakup time of tear film (TBUT) ≤ 5 s, (6) Schirmer test>5 s, (7) Meibo-Scan (OCULUS) revealed less than 1/3 atrophy area of the meibomian gland in both the upper and lower eyelids, (8) refractory was defined as lack of symptom relief with conservative treatment (eyelid warming, massage, and artificial tears) for at least 1 year prior to study treatment. All patients were informed of possible treatment-related complications and the possibility of being assigned to an invasive treatment group. All agreed to receive the possible therapeutic regimen and signed an informed consent form. Patients with a history of corneal contact lens, mite blepharitis, acute eye inflammation, or infection and apparent eyelid margin scarring as well as patients using a lacrimal plug or receiving LASIK (Laser Assisted In-situ Keratomi) were excluded from the study.

The multiple rate comparison method performed with PASS version 15 was used to estimate sample size. The pilot study, which involved 5 patients per group, showed that 20, 20, and 80% of patients in IPL, MGP and MGP-IPL groups experienced effective symptom improvement following treatment (with a decrease in SPEED score before treatment and half a year after final treatment>5). Power calculations with a type I error of 0.05 and type II error of 0.9 were executed. The results showed a sample size of 38 achieves 90% power in detecting an effect size (W) of 0.5774 using a 2 degrees of freedom Chi-Square Test with a significance level (alpha) of 0.05. So, each group needed at least 13 patients.

Participants were randomly divided into 3 groups (15 patients per group) via block randomization, and allocation concealment was implemented using a closed envelop method. Patients in group I received an IPL treatment course (treated with IPL 3 times at 3-week intervals). Patients in group II received an MGP treatment course (treated with MGP one time). In group III, 3 weeks after initial MGP treatment, patients also received IPL 3 times at 3-week intervals. The clinical effects were assessed at baseline, 3 and 12 weeks following MGP treatment for group II and 3 weeks after final treatment for groups I and III. Furthermore, 6 months following final treatment for all 3 groups, all patients completed SPEED and answered a question in terms of requiring to receive any treatment once more. Patient enrollment, random allocation sequence generation, and intervention assignment were performed by the first author (HXD).

### Treatment procedure

#### Intraductal meibomian canal probing

**Fig. 1:**
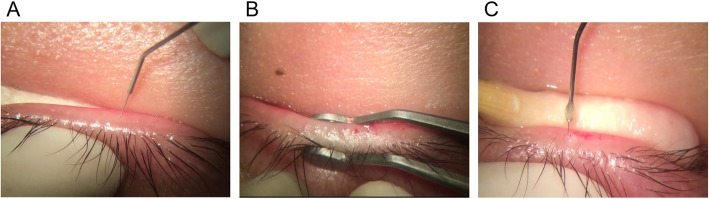
The treatment procedure and structure of our private probe and rinse hollow tube. **a** the operator inserted the probe into the glands vertically to the orifices. **b** After probing, chalazion forceps were used to squeeze out remnant meibum. **c** Then, a hollow tube was used to swash the meibomian gland by injecting

With the help of SuZhou LiuLiu Medical Equipment co. LTD, we designed a private probe based on the original Maskin probe and a rinse hollow tube (Fig. 1). The probe was 4.5 mm in length with a blunt end 0.12 mm in diameter. The hollow tube was 2.0 mm in length and 0.16 mm in diameter. The process of intraductal MGP proceeded as follows: (1) to ease the pain of probing, 4% lidocaine was injected into the upper and lower eyelids parallel to the palpebral margin, resulting in a local bulgy of the skin. (2) the eyelids were flipped outward with a cotton swab and an operating microscope was positioned over the target eyelid to more clearly show the orifices. Then, the operator inserted the probe into the glands vertically to the orifices. Impact force was required when resistance from the orifices or intraductal was encountered. After probing, chalazion forceps were used to squeeze out remnant meibum. Self-limited hemorrhage was the most common complication, for which a blood point and blood trickle could be observed and no particular treatment was needed. (3) then, a hollow tube was used to swash the meibomian gland by injecting 0.1% Dexamethasone (Guangzhou Baiyun Mountain Pharmaceutical co. LTD, China) and 0.25% Amikacin (Qilu Pharmaceutical co. LTD, China) repeatedly (Fig. 1). (4) eventually, Tobradex eye ointment (Alcon, Belgium) was applied to the conjunctival sac. All MGP procedures were performed by the first author (HXD).

#### Intense pulsed light

A M22 Multi-pulse therapeutic apparatus was used for treatment. Prior to treatment, 1–2 mm thick ultrasound gel was applied to participants’ faces, covering the area from tragus to tragus beneath the eyelid margin, temple, and forehead. Then, the Pre-set Toyos parameters were administered to 1 or 2 treatment area test points to test patient tolerance and comfort. The intensity of the IPL treatment was adjusted to 14 J/cm^2^-15 J/cm^2^, which was determined via Fitzpatrick Skin Type Grading. Placement of an IPL eye shield over the eyes was necessary to protect eyes from the stimulus of bright light. After this, one back-and-forth flash emitted by an IPL hand piece was placed on each skin area without pressure. Finally, chalazion forceps were used to squeeze MG tissues. Care should be taken to ensure that the treatment areas were identical for each participant and all procedures were conducted by the same doctor (LL).

All participants were required to use artificial tears (Hailu, German) four times a day during the entire follow-up period.

### Clinical evaluation

The eye examiners (Jiao Zheng and Linping Wang) were blind in regard to the groups participants were assigned to.

#### SPEED, CFS and TBUT

A Standard Patient Evaluation of Eye Dryness (SPEED) validated questionnaire (0–28) was used to assess the symptoms, as previously described [15]. Corneal fluorescein staining (CFS) was evaluated by dividing the cornea into four equal quadrants, and the staining of each section was recorded on a 0–3 scale: 0 = no punctate staining; 1 = less than half staining; 2 = more than half staining; 3 = whole staining; and a composite score for each quadrant (0–12 score) [16]. Tear break-up time (TBUT) was evaluated 3 times and an average value was recorded [17].

#### Meibum grade

The lower and upper eyelids were divided into 3 parts– nasal, bitamporal, and middle– with a total of 15 glands in each eyelid. The characteristics of each glandular expressate were graded on a scale of 0 to 3: 0 = no secretion; 1 = inspissated-filamentary secretion; 2 = cloudy liquid secretion; and 3 = clear liquid secretion. The scores of each expressed orifice in the 3 different eyelid sections were added together to provide the final meibum grade scores (0–90 score) for the right and left eyes [18].

#### Lid margin finding results

Lid margin finding results we evaluated included the abnormality of meibomian gland orifices, lid tenderness and telangiectasia, and were noted on a 0–4 scale, with 0 being absent and 4 being the most severe [8, 19].

### Statistical analysis

Statistical significance was set at *p* < 0.05, and data analysis was performed using SPSS version 23. Continuous data was presented as means ± SD. A paired Wilcoxon test was employed to compare the parameters prior to and following treatment. Then, comparison was made between the different groups via non-parametric Mann-Whitney U tests with Bonferroni correction, Kruskal-Wallis tests.

## Results

A total of 45 patients were at first enrolled in the study, with one patient in the IPL group ending the treatment course due to accidental pregnancy and one patient in the MGP-IPL group for home accidents. The ages of 43 enrolled patients (86 eyes) ranged from 24 to 56 years (mean age 37.56 ± 9.82), with a female to male ratio of 1.39. And there were no observed differences based on gender (*P* = 0.409) and age (*P* = 0.376) among the 3 groups.

During the follow-up period, several MGP-treated patients experienced subcutaneous ecchymosis of the eyelid skin caused by the injection of anesthetics, a symptom that can improve after the administration of a cold compress. And one patient in the IPL group suspended the treatment course due to occurred blepharokeratoconjunctivtis (BKC) after twice IPL treatments, with the final IPL not being performed until BKC was relieved via two-week administration of Tobradex.

The evaluation time for the MGP group was 3 and 12 weeks following MGP treatment, but no difference in all indexes was found to exist between 3 and 12 weeks after MGP treatment (SPEED: 11.87 ± 3.44 vs. 11.93 ± 3.26, *P* = 0.933; TBUT: 4.74 ± 1.28 vs. 4.81 ± 2.03, *P* = 0.539; CFS: 0.73 ± 1.34 vs. 0.80 ± 1.35, *P* = 0.801; meibum grade: 24.73 ± 10.66 vs. 26.57 ± 11.63, *P* = 0.534; lid telangiectasia: 1.73 ± 0.58 vs. 1.73 ± 0.64, *P* = 0.946; orifice abnormality: 2.00 ± 0.74 vs. 1.80 ± 0.85, *P* = 0.299; lid tenderness: 0.60 ± 0.67 vs. 0.57 ± 0.63, *P* = 0.901). In order to increase the comparability of the MGP and MGP-IPL groups (both assessed at 12 weeks after initial MGP treatment), the 12-week-data for the MGP-treated group II was selected as posttreatment data for analysis.

Prior to initial treatment, there were no observed differences among all parameters of the 3 groups (SPEED: *P* = 0.339; TBUT: *P* = 0.083; CFS: *P* = 0.517; meibum grade: *P* = 0.139; lid telangiectasia: *P* = 0.105; orifice abnormality: *P* = 0.180; lid tenderness: *P* = 0.175). After completion of the entire treatment course, all subjective symptoms and objective signs, including SPEED, TBUT, CFS, meibum grade, lid telangiectasia, orifice abnormality, and lid tenderness, were significantly improved for all groups (Table.1).

**Table 1:** Clinical parameters before and after treatment in refractory O-MGD patients

	Group I (IPL)		P	Group II (MGP)		P	Group III (MGP-IPL)		P
Scores	before	after		before	after		before	after	
SPEED	16.14 ± 3.53	12.43 ± 3.84	<0.001	17.13 ± 3.23	11.93 ± 3.26	<0.001	18.00 ± 3.51	9.00 ± 1.80	<0.001
TBUT	2.66 ± 0.88	4.35 ± 0.88	<0.001	3.21 ± 0.98	4.81 ± 2.03	<0.001	2.78 ± 1.00	6.61 ± 1.57	<0.001
CFS	2.29 ± 2.71	0.96 ± 2.10	<0.001	2.13 ± 2.34	0.80 ± 1.35	<0.001	2.79 ± 2.51	0.29 ± 0.71	<0.001
Meibum grade	7.11 ± 4.57	20.82 ± 11.83	0.003	8.23 ± 3.15	26.57 ± 11.63	<0.001	6.64 ± 3.41	41.11 ± 10.26	<0.001
Lid telangiectasia	2.36 ± 0.49	1.43 ± 0.50	0.006	2.27 ± 0.45	1.73 ± 0.64	0.001	2.54 ± 0.51	1.07 ± 0.26	0.001
Orifice abnormality	2.14 ± 0.52	1.54 ± 0.51	<0.001	2.30 ± 0.60	1.80 ± 0.85	<0.001	2.00 ± 0.67	1.29 ± 0.46	<0.001
Lid tenderness	1.79 ± 0.79	1.36 ± 0.49	0.003	2.13 ± 0.57	0.57 ± 0.63	0.001	1.93 ± 0.81	0.36 ± 0.49	<0.001

*P* values were determined with a paired Wilcoxon test

“AFTER” was determined as 3 weeks after final treatment for groups I and III and 12 weeks after final treatment for group II

**Fig. 2:**
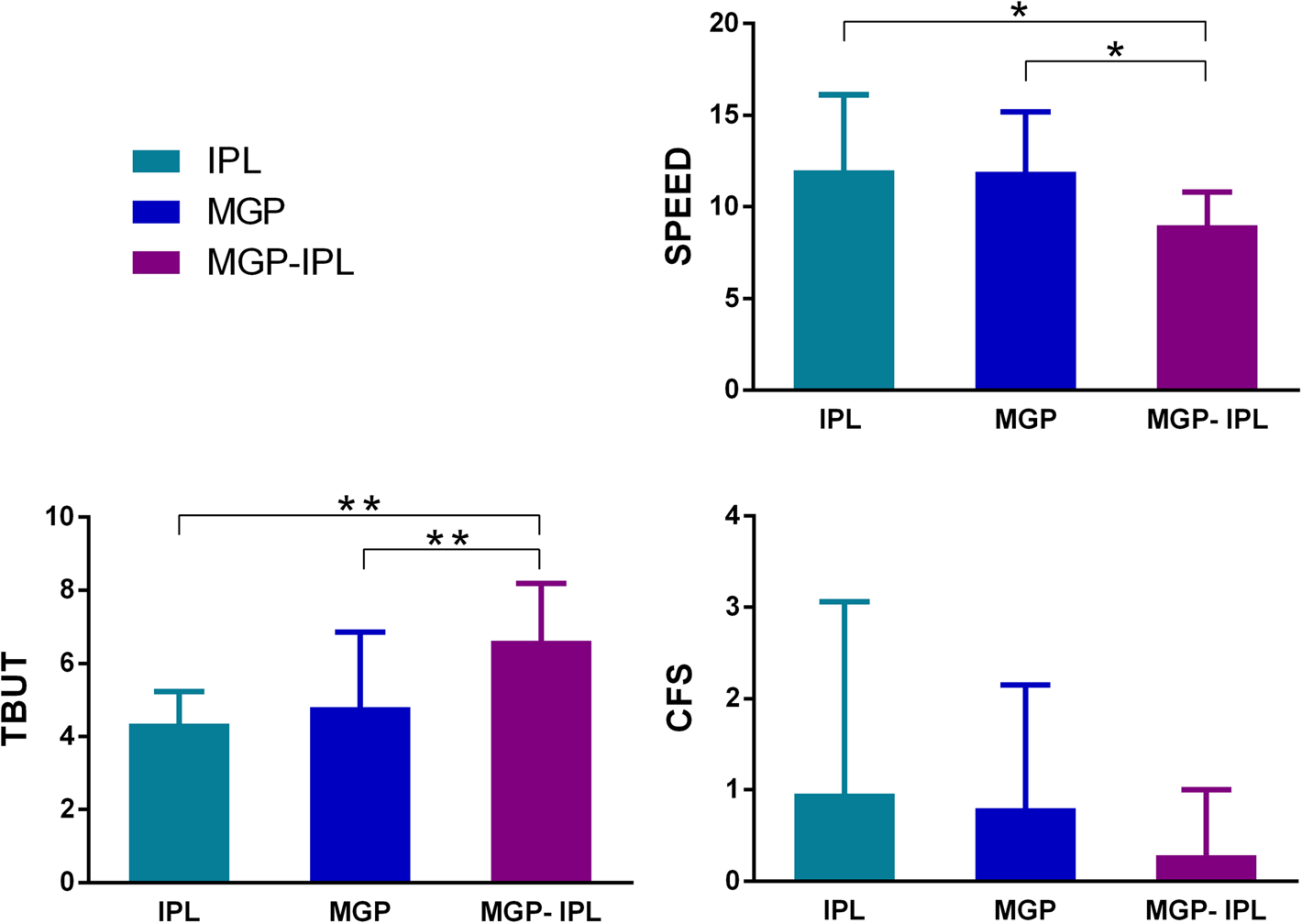
Comparation of SPEED score, TBUT and CFS after treatment in 3 groups (IPL, MGP, MGP-IPL). Notes: all parameters prior treatment had no statistical differences among 3 groups. *P ≤ 0.05/3, **P<0.001; “AFTER” was determined as 3 weeks after final treatment for groups I and III and 12 weeks after final treatment for group II, the same below

The improvement of ocular symptoms (SPEED) and TBUT was more apparent in the MGP-IPL group than the IPL and MGP groups (*P* = 0.003 or *P* = 0.012; Fig. 2). However, there were no observed differences in posttreatment CFS among 3 groups (group IPL vs. group MGP, *P* = 0.866; group IPL vs. group MGP-IPL, *P* = 0.084; group MGP vs. group MGP-IPL, *P* = 0.123; Fig. 2). Between group IPL and group MGP, no differences existed in SPEED, TBUT, CFS after treatment (SPEED: *P* = 0.339; TBUT: *P* = 0.083; CFS: *P* = 0.517; Fig. 2).

**Fig. 3:**
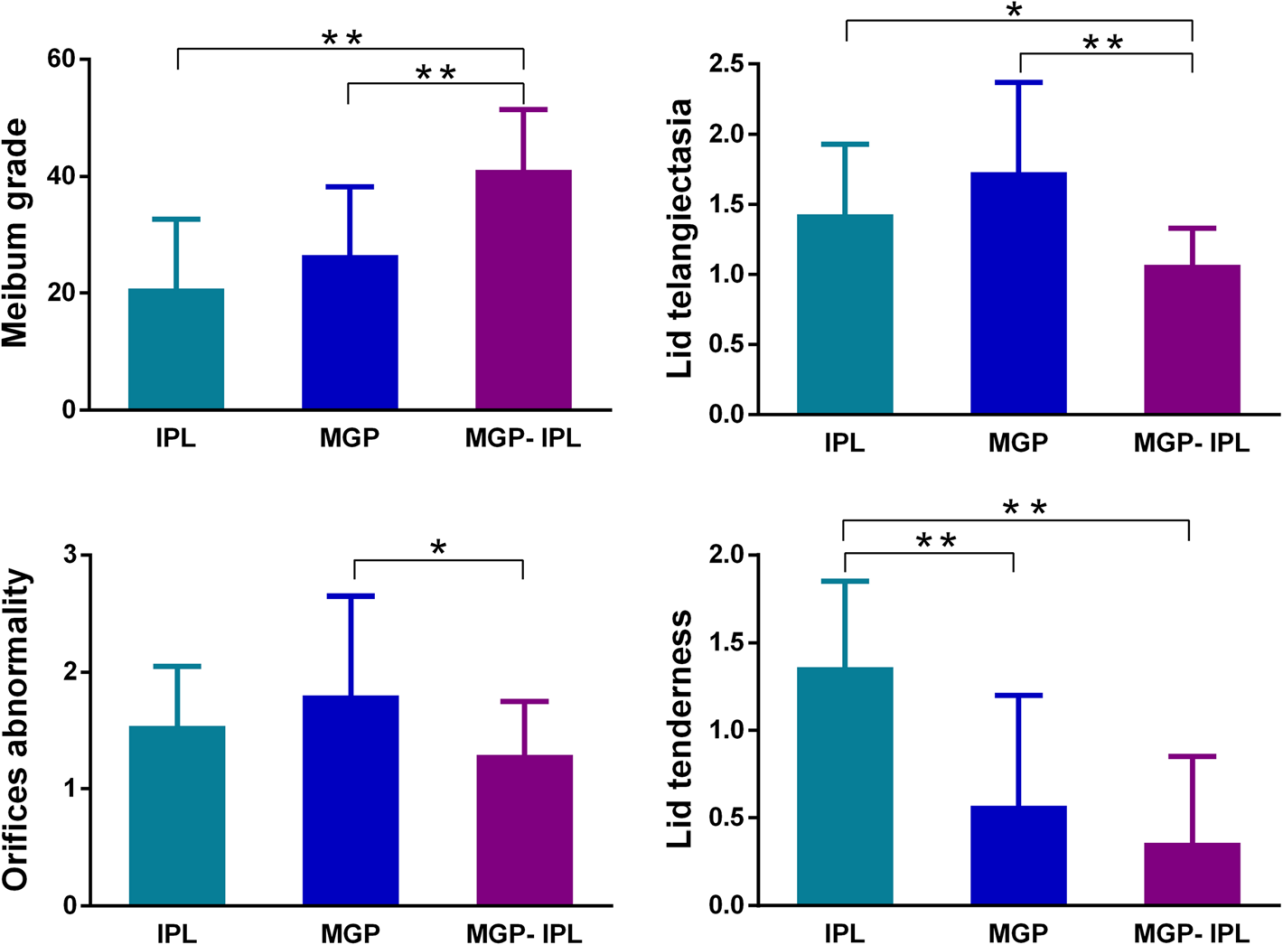
Comparation of meibum grade and lid margin finding results after treatment in 3 groups. Notes: all parameters prior treatment had no statistical differences among 3 groups. *P ≤ 0.05/3, **P<0.001

As for lid margin related indexes, the posttreatment meibum grade and lid telangiectasia improved more for group MGP-IPL than group IPL or group MGP (*P* = 0.002 or P<0.001, respectively; Table.1, Fig. 3). Orifice abnormality after treatment was also significantly more improved for the MGP-IPL group than the MGP group (*P* = 0.016; Table.1, Fig. 3). In terms of lid tenderness, group MGP-IPL showed more significant improvement than group IPL (*P*<0.001; Table.1, Fig. 3). No differences in meibum grade, lid telangiectasia, and orifice abnormality were observed among group IPL and group MGP (meibum grade: *P* = 0.040; lid telangiectasia: *P* = 0.068; orifices abnormality: *P* = 0.315; Fig. 3) except for lid tenderness, in which better results were seen in group MGP (P<0.001; Table.1, Fig. 3).

**Fig. 4:**
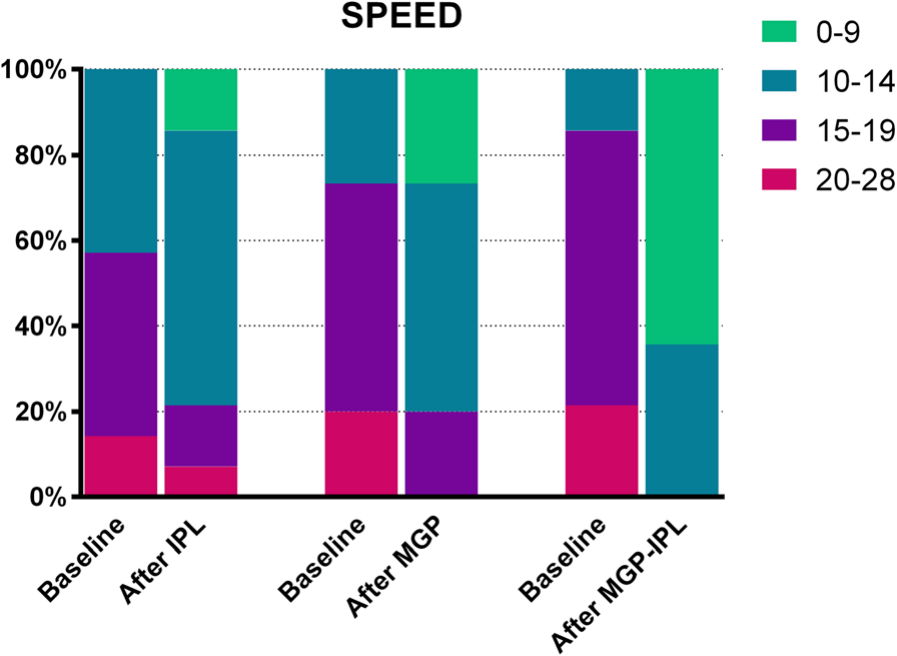
Change in the SPEED questionnaire score between baseline and after treatment in three groups

**Fig. 5:**
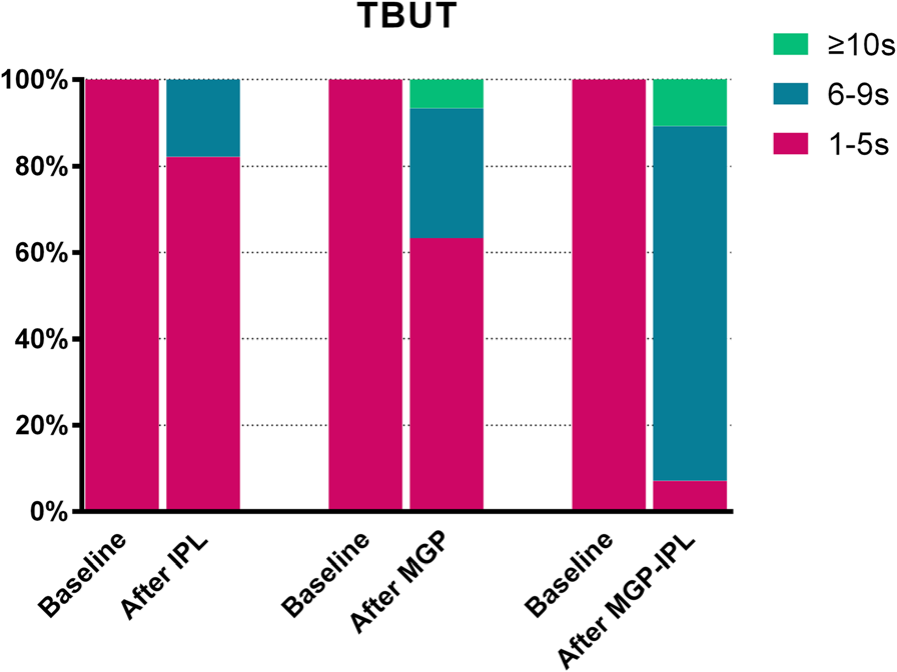
Change in TBUT between baseline and after treatment in three groups

As shown in Fig. 4, no patient from any group displayed a SPEED score ≤ 9 before treatment; while following treatment, 14.29, 26.67, and 64.29% of patients in groups I, II, and III, respectively, obtained a score of 0–9 (*P* = 0.020, P was determined by the Fisher exact test). Moreover, it can be seen that all eyes in 3 groups showed a TBUT≤5 s before treatment, but 17.86, 36.67, and 92.9% of eyes in group I, II, and III, respectively, showed a TBUT more than 5 s after treatment (*P* = 0.009, χ2 = 7.335, P was determined by χ2 test; Fig. 5).

Six months after final treatment, the SPEED was still significantly lower in patients receiving MGP-IPL than MGP or IPL alone (11.36 ± 2.10 vs. 14.50 ± 3.76 vs. 14.60 ± 3.11, *P* = 0.01 or *P* = 0.004). Additionally, 35.7% or 20% of patients treated with IPL or MGP alone reported requiring treatment again to rectify recurrent dry eye related symptoms; meanwhile, of the patients who received the combined MGP-IPL course, zero reported a need to be treated again.

## Discussion

Previous research has proven that both intraductal meibomian gland probing and intense pulsed light are significantly efficient in helping o-MGD patients achieve relief of symptoms and signs; yet, they also showed that this improvement was only experienced by the majority and symptom recurrence could emerge during the follow-up period [13]. Until now, no research has offered in-depth discussion for these exceptions. It seems researchers all focused on the pleasantly impressive results of these new treatments, but seldom noticed their inadequacies. Although MGP can re-open MG orifices, it is limited in terms of controlling inflammation. Moreover, it is an invasive treatment, so the repeated use of MGP should be restricted. IPL treatment is minimally invasive and can promote the discharge of eyelid lipids, reducing the inflammation of the eyelid margin. However, the effect of IPL on MG-obstruction and scarring is limited. Therefore, a new treatment combination that could fully realize the best therapeutic effects of two treatments and reduce the complications of invasive probing is essential.

Reiko Arita et al. recently observed that 81% of IPL-treated refractory o-MGD eyes showed amelioration of ocular symptoms, and 70% showed an improvement in TBUT [20]. Zeba A et al. reported that 91.4% of their patients received MGP described subjective symptomatic improvement during follow-up [21]. Similar results were also obtained in the present study, with 85.7 and 100% of treated eyes in the IPL and MGP groups revealing relief of symptoms, and 96.4 and 93.3% exhibiting increase in TBUT, respectively. However, in the MGP-IPL group, all patients (100%) showed alleviation of dry eye related symptoms as well as the extension of TBUT.

As the meibomian gland of an o-MGD patient is usually ill-conditioned, in which abnormal meibum stasis accumulates rather than flows to the ocular surface, increased intraglandular pressure and duct expansion are inevitable [14]. Furthermore, with the recurrent attacks of o-MGD, atrophy of meibomian glands is frequently observed [22]. It was long considered that this atrophy was irreversible until Maskin proposed intraductal meibomian gland probing and proved this treatment can increase MG tissue area and growth of atrophied MGs [12, 22]. Maskin showed that they used transillumination to ensure the gland was longer than the length of the probe before probing. Their most common length of probe was 4 mm. And they showed their probes can probe to the most distal aspects of the duct [12]. Our private probe was 4.5 mm in length, and before probing, we used infrared meibography (IR-M) to know the length of glands, so we believe that our MGP treatment is also enable to affect far distal part of meibomian gland to reopen the blocked sites effectively. Meibomian gland probing mechanically opened the obstructed orifices and ducts. With the pop up of constrained meibum, keratinized epithelium, and debris, the vicious cycle of o-MGD progression was broken, and the majority of patients received immediate symptom relief [10, 21]. However, the quantity of meibum on the ocular surface is not a decisive factor in the retardation of the evaporation of aqueous and the stabilization of the tear film. The meibum lipid quality was found to play an even more important role in maintaining ocular surface equilibrium [14, 23]. Nakayama et al. showed all cases exhibited improvements in meibum viscosity (grades 3–0, 3–1, and 3–2) after MGP treatment, as the abnormal meibum was rapidly released with the sudden orifice opening and then gradually eliminated through blinking [14]. However, there was only one case returning to normal level. Furthermore, a growing amount of evidence has suggested the inflammation reaction played an essential role in the formation of abnormal meibum. The enzymes produced by bacterial flora could result in altered lipid composition with an increased melting point and viscosity [3, 24]. Thus, it was assumed that the single mechanical function of MGP in improving meibum lipid quality is limited. Xiao Ma et al. recommended the use of 0.1% fluorometholone after MGP treatment to diminish inflammation, since MGP predisposes the lid margin to a topical corticosteroid effect [10]. However, it is believed that although MGP increased the responsiveness of the gland to anti-inflammatory drugs, the traditional application of eyedrops or eye ointment following MGP can hardly deliver drugs to the deepest gland lumens. Since the inflammation of o-MGD has been proven to not only exist in the eyelid margin and ocular surface but also within the glands [25], the unthorough evacuation of inflammation after MGP treatment may be essential for the re-obstruction, possibly explaining why not all patients experienced improvement after MGP treatment and why a considerable number of patients needed to receive repeated probing.

The surprising efficacy of IPL in easing the symptoms of MGD patients can be mainly attributed to its effect of vasculature destruction and meibum melting [26, 27]. Lid telangiectasia is a common characteristic of o-MGD, and these tiny vessels along the eyelid margin also increase the accessibility of inflammatory mediators, resulting in aggravated chronic inflammation above the palpebral edge or within the glands [28–30]. The 580 nm wavelength released by intense pulsed light can be absorbed by intravascular hemoglobin and then activate selective photothermolysis, leading to the development of blood clotting. Thus, abnormal vessels gradually shut down and bacterial loading reduces [26]. Apart from that, the heat from either photothermolysis or light energy itself can enhance the liquidity of meibum. And compared to traditional eyelid warming, the heat effect delivered by intense pulsed light is far more lasting and permeable [31]. Surprisingly, instead of showing reduction in symptoms, 2 patients (14.8%) in the present study reported even more serious symptoms at the end of the IPL treatment course. It can be speculated that this deterioration may relate to obstruction sites within the glands. Maskin has proposed six types of o-MGD according to the depths of fixed obstruction and the function of MG [22]. In a meibomian gland with a deep-seated intratubal obstruction or partial distal obstruction, IPL may work well as the vast melting meibum ahead the fixed area can easily move out under the extrusion force caused by forceps or daily blinking. While for the gland that was completely fixed in the distal part, it’s actually the opposite, as the stagnant meibum was confined between the terminal of glands and the obstruction site, analogous to staying in a blind alley. The heat released by IPL and the pressure caused by the forceps might paradoxically increase the intraductal pressure and exacerbate the inflammatory response; thus, treatment with IPL alone may not alleviate disease symptoms but instead irritate the condition. This effect can also be indirectly observed in the present data in terms of the posttreatment lid tenderness of the IPL group, despite showing symptom alleviation compared with baseline, still being significantly higher than the MGP and MGP-IPL groups.

It appears that neither IPL nor MGP is the absolute perfect method for treating all refractory o-MGD patients; however, their unique advantages can effectively make up for their inherent deficiencies. This assumption was also confirmed by the present research, as patients receiving MGP-IPL treatment exhibited the best improvement results. With the initial opening of blocked glands via probing, meibum within the glands can flow without restriction. Additionally, the followed 3 times IPL treatments further restrict inflammation and eliminate the abnormal meibum, resulting in an optimal therapeutic effect. Compared with single IPL or MGP treatment, MGP combined IPL proved to be significantly superior in improving SPEED, TBUT, meibum grade, and lid telangiectasia.

One time MGP did not provide all patients continued symptom relief in the present 6-month observation. Specifically, 20% of patients still required repeated invasive probing, yet such treatment would increase patients’ sense of misery. In contrast, the combination of MGP with noninvasive IPL in the present study helped 100% of patients attain enduring symptom relief. This combination treatment may achieve the maximum therapeutic effect of MGP and IPL, reducing the possibility of trauma and scarring caused by repeated probing.

Despite positive outcomes, there are still certain limitations of the present research: First, the participants in the study were comparatively small and the follow-up duration was rather short. Further investigation is thus suggested to evaluate the long-term results of these treatments with a larger number of cases. Second, MGP is an invasive method that is more suitable for patients with severe gland obstruction or gland scarring, while IPL treatment is better for relieving intraductal inflammation. This study found the combination of these two treatments could attain the best results, but it cannot be denied that this treatment mode would bring patients more financial, time and psychological burdens at the same time. Based on these results, it is recommended that patients have at least half of their orifices obstructed in each eyelid but with no apparent meibomian gland atrophy, and at the same time, have higher inflammatory index like lid telangiectasia scores receive combined MGP-IPL therapy to exert the best curative effect of probing and anti-inflammation simultaneously.

## Conclusions

IPL, MGP, and combined MGP-IPL are all effective methods for refractory o-MGD patients; however, the combination MGP-IPL method could maximize the therapeutic benefits, which is especially helpful for patients who have severe meibomian gland obstruction and obvious intraductal or eyelid margin inflammation, who want to gain the greatest amelioration in all clinical signs and subjective symptoms or still remain frustrated to either MGP or IPL treatment.

## Data Availability

The datasets obtained and/or analyzed during the current study are available from the corresponding author on reasonable request.

## Abbreviations

BKC: Blepharon- keratoconjunctivtis
CFS: Corneal fluorescein staining
IPL: Intense pulsed light
IR-M: infrared meibography
LASIK: Laser Assisted In-situ Keratomi
MG: Meibomian gland
MGP: Intraductal meibomian gland probing
o-MGD: Obstructive meibomian gland dysfunction
SPEED: Standard Patient Evaluation of Eye Dryness score
TBUT: Tear break-up time

## References

[1] Waduthantri S, Yong SS, Tan CH (2012). Cost of dry eye treatment in an Asian clinic setting. PLoS One.

[2] Miljanovic B, Dana R, Sullivan DA, Schaumberg DA (2007). Impact of dry eye syndrome on vision related quality of life. Am J Ophthalmol.

[3] Nelson JD, Shimazaki J, Benitez-del-Castillo JM (2011). The international workshop on meibomian gland dysfunction: report of the definition and classification subcommittee. Invest Ophthalmol Vis Sci.

[4] Rabensteiner DF, Aminfar H, Boldin I, Schwantzer G, Horwath-Winter J. The prevalence of meibomian gland dysfunction, tear film and ocular surface parameters in an Austrian dry eye clinic population. Acta Ophthalmol. 2018. 10.1111/aos.13732. 10.1111/aos.13732 PMC6619403 29656524

[5] Foulks GN, Bron AJ. Meibomian gland dysfunction: a clinical scheme for description, diagnosis,classification, and grading. Ocul Surf 2003;1:107–126. 10.1016/s1542-0124(12)70139-8 17075643

[6] Gayton JL (2009). Etiology, prevalence, and treatment of dry eye disease. Clin Ophthalmol.

[7] Goto E, Monden Y, Takano Y (2002). Treatment of non-inflamed obstructive meibomian gland dysfunction by an infrared warm compression device. Br J Ophthalmol.

[8] Ma X, Lu Y (2016). Efficacy of Intraductal Meibomian gland probing on tear function in patients with obstructive Meibomian gland dysfunction. Cornea..

[9] Arita R, Morishige N, Koh S, Shirakawa R, Kawashima M, Sakimoto T (2015). Increased tear fluid production as a compensatory response to Meibomian gland loss: a multicenter cross-sectional study. Ophthalmology..

[10] Gupta PK, Vora GK, Matossian C, Kim M, Stinnett S (2016). Outcomes of intense pulsed light therapy for treatment of evaporative dry eye disease. Can J Ophthalmol.

[11] Jiang X, Lv H, Song H (2016). Evaluation of the safety and effectiveness of intense pulsed light in the treatment of Meibomian gland dysfunction. J Ophthalmol.

[12] Maskin SL (2010). Intraductal meibomian gland probing relieves symptoms of obstructive meibomian gland dysfunction. Cornea..

[13] Sik Sarman Z, Cucen B, Yuksel N, Cengiz A, Caglar Y (2016). Effectiveness of Intraductal Meibomian gland probing for obstructive Meibomian gland dysfunction. Cornea..

[14] Nakayama N, Kawashima M, Kaido M, Arita R, Tsubota K (2015). Analysis of Meibum before and after Intraductal Meibomian gland probing in eyes with obstructive Meibomian gland dysfunction. Cornea..

[15] Asiedu K, Kyei S, Mensah SN, Ocansey S, Abu LS, Kyere EA (2016). Ocular surface disease index (OSDI) versus the standard patient evaluation of eye dryness (SPEED): a study of a nonclinical sample. Cornea..

[16] Song X, Zhao P, Wang G, Zhao X (2014). The effects of estrogen and androgen on tear secretion and matrix metalloproteinase-2 expression in lacrimal glands of ovariectomized rats. Invest Ophthalmol Vis Sci.

[17] Lee H, Kim M, Park SY, Kim EK, Seo KY, Kim TI (2017). Mechanical meibomian gland squeezing combined with eyelid scrubs and warm compresses for the treatment of meibomian gland dysfunction. Clin Exp Optom.

[18] Greiner JV (2016). Long-term (3 year) effects of a single thermal pulsation system treatment on Meibomian gland function and dry eye symptoms. Eye Contact Lens.

[19] Jin X, Lin Z, Liu Y, Lin L, Zhu B (2016). Hormone replacement therapy benefits meibomian gland dysfunction in perimenopausal women. Medicine (Baltimore).

[20] Arita R, Mizoguchi T, Fukuoka S, Morishige N. Multicenter study of intense pulsed light therapy for patients with refractory Meibomian gland dysfunction. Cornea. 2018. 10.1097/ico.0000000000001687. 10.1097/ICO.0000000000001687 PMC6221410 30004962

[21] Syed ZA, Sutula FC (2017). Dynamic Intraductal Meibomian probing: a modified approach to the treatment of obstructive Meibomian gland dysfunction. Ophthal Plast Reconstr Surg.

[22] Maskin SL, Testa WR (2018). Growth of meibomian gland tissue after intraductal meibomian gland probing in patients with obstructive meibomian gland dysfunction. Br J Ophthalmol.

[23] Ashraf Z, Pasha U, Greenstone V, Akbar J, Apenbrinck E, Foulks GN (2011). Quantification of human sebum on skin and human meibum on the eye lid margin using Sebutape(R), spectroscopy and chemical analysis. Curr Eye Res.

[24] Lee H, Chung B, Kim KS, Seo KY, Choi BJ, Kim TI (2014). Effects of topical loteprednol etabonate on tear cytokines and clinical outcomes in moderate and severe meibomian gland dysfunction: randomized clinical trial. Am J Ophthalmol.

[25] Liu S, Richards SM, Lo K, Hatton M, Fay A, Sullivan DA (2011). Changes in gene expression in human meibomian gland dysfunction. Invest Ophthalmol Vis Sci.

[26] Toyos R, McGill W, Briscoe D (2015). Intense pulsed light treatment for dry eye disease due to meibomian gland dysfunction; a 3-year retrospective study. Photomed Laser Surg.

[27] Dell SJ, Gaster RN, Barbarino SC, Cunningham DN (2017). Prospective evaluation of intense pulsed light and meibomian gland expression efficacy on relieving signs and symptoms of dry eye disease due to meibomian gland dysfunction. Clin Ophthalmol.

[28] Bron AJ, Benjamin L, Snibson GR (1991). Meibomian gland disease. Classification and grading of lid changes. Eye.

[29] Geerling G, Tauber J, Baudouin C (2011). The international workshop on meibomian gland dysfunction: report of the subcommittee on management and treatment of meibomian gland dysfunction. Invest Ophthalmol Vis Sci.

[30] Shine WE, McCulley JP (2003). Polar lipids in human meibomian gland secretions. Curr Eye Res.

[31] Arita R, Morishige N, Shirakawa R, Sato Y, Amano S (2015). Effects of eyelid warming devices on tear film parameters in Normal subjects and patients with Meibomian gland dysfunction. Ocul Surf..

